# Longitudinal associations of diet quality with serum biomarkers of lipid and amino acid metabolism from childhood to adolescence: the PANIC study

**DOI:** 10.1017/S0007114525000492

**Published:** 2025-04-14

**Authors:** Suvi E. Laamanen, Saara Heinonen, Eero A. Haapala, Ursula Schwab, Sonja Soininen, Taisa Sallinen, Aino-Maija Eloranta, Timo A. Lakka

**Affiliations:** 1 Institute of Public Health and Clinical Nutrition, School of Medicine, University of Eastern Finland, Kuopio, Finland; 2 Institute of Biomedicine, School of Medicine, University of Eastern Finland, Kuopio, Finland; 3 Faculty of Sport and Health Sciences, University of Jyväskylä, Jyväskylä, Finland; 4 Department of Medicine, Endocrinology and Clinical Nutrition, Kuopio University Hospital, Wellbeing Services County of North Savo, Kuopio, Finland; 5 Physician, and Nursing Services, Health and Social Services Centre, Wellbeing Services County of North Savo, Varkaus, Finland; 6 Department of Clinical Physiology and Nuclear Medicine, Kuopio University Hospital, Finland; 7 Kuopio Research Institute of Exercise Medicine, Kuopio, Finland

**Keywords:** Diet quality, Food consumption, Metabolites, Children, Adolescents

## Abstract

Studies on longitudinal associations between diet quality and lipid and amino acid metabolism in children and adolescents are limited. We studied associations between diet quality and serum markers of lipid and amino acid metabolism in the Physical Activity and Nutrition in Children (PANIC) study. These analyses included 403 children aged 6–9 years at baseline, 360 re-examined 2 years later at age 9–11 years and 219 eight years later at age 15–17 years. Food intake was recorded over 4 days, and diet quality was assessed using the Finnish Children Healthy Eating Index (FCHEI). Fasting serum fatty acids, amino acids, apolipoproteins and lipoprotein particle sizes were analysed via NMR spectroscopy. Linear mixed-effects models, adjusted for sex, age, body fat percentage, pubertal stage and physical activity were used to analyse the associations. Better diet quality was linked to increased serum PUFA and reduced saturated and MUFA, alanine and VLDL particle size. Consuming more vegetables, fruits, berries, vegetable oils and margarine with at least 60 % fat, fish and whole grains is associated with higher serum PUFA, lower SFA and smaller VLDL particles. Conversely, consuming higher-fat dairy products and sugary products is associated with higher saturated and MUFA, branched-chain and aromatic amino acids and larger VLDL particles. A diet rich in fruits, vegetables, unsaturated fats and fibre, with reduced sugar consumption, promotes favourable metabolic changes relevant to cardiometabolic health.

The burden of non-communicable diseases (NCD), such as obesity, type 2 diabetes and CVD, has increased globally^(1,2)^. Although NCD exhibit their clinical manifestations in adulthood, the pathophysiological processes leading to these diseases can begin at a very young age^(3–6)^. Lower diet quality has been recognised as an underlying risk factor for NCD^(7,8)^. Furthermore, several serum metabolites, small molecules resulting from metabolic processes in the body, have been associated with both lower diet quality and an increased risk of NCD in adults^(9)^. For instance, higher serum concentrations of branched-chain amino acids (BCAA) and SFA as well as larger VLDL particles have been linked to higher blood pressure, insulin resistance and CVD in cross-sectional studies in adults^(9,10)^. Moreover, higher serum levels of some diet-associated metabolites, such as BCAA, have been related to risk factors for NCD already in children^(11)^. These findings raise curiosity about the role of blood metabolites as mediating factors between diet and NCD and whether these effects are seen already in childhood.

Nutrition and metabolomics research in children and adolescents has largely focussed on the associations of dietary factors with blood fatty acids^(12–18)^. Interestingly, we previously observed that better diet quality, characterised by a higher intake of dietary sources of PUFA and fibre and a lower intake of sugary products, was reflected in a more unsaturated serum fatty acid profile, a larger VLDL particle size and lower serum levels of alanine, histidine and glycine in children^(19)^. Our results suggest that diet quality might influence blood levels of various metabolites related to cardiometabolic health^(9,10)^, thus also underlining the importance of studying a wide range of blood metabolites rather than focusing on only a limited number of biomarkers to provide a more comprehensive view. Additionally, in Finnish children and adolescents, the consumption of vegetables, fruit and berries is low and the consumption of dietary SFA and sucrose is over the recommendations of the Finnish dietary guidelines^(20–22)^. These results also highlight the importance of studying the association between diet quality and metabolites in a population sample of Finnish children and adolescents. However, only a few reports on the longitudinal associations of dietary factors with circulating metabolites of amino acid and lipid metabolism in children and adolescents have been published^(23,24)^. The results of these reports, which were based on the same Finnish dietary intervention study, showed that better adherence to Nordic nutritional recommendations was reflected in higher serum levels of PUFA, lower serum levels of SFA and a larger LDL particle size. This existing, yet scarce, evidence emphasises the need for further longitudinal studies examining the associations of diet with blood metabolites from childhood to adolescence.

Given the limited evidence on the longitudinal associations of dietary factors with blood metabolites in youth, we investigated the associations of diet quality with serum metabolites in a general population of children followed up for 8 years until adolescence. We hypothesised that good diet quality is associated with serum levels of metabolites considered beneficial for cardiometabolic health, such as higher PUFA and lower BCAA.

## Methods

### Study design and participants

These analyses on the longitudinal associations of diet quality with serum biomarkers of lipid and amino acid metabolism from childhood to adolescence are based on the baseline, 2-year and 8-year data of the Physical Activity and Nutrition in Children (PANIC) study, which is an 8-year physical activity and diet intervention and long-term follow-up study in a population sample of children from the city of Kuopio, Finland. The PANIC study protocol was approved by the Research Ethics Committee of the Hospital District of Northern Savo in 2006 (Statement 69/2006) and in 2015 (Statement 422/2015). The parents or caregivers gave their written informed consent, and the children provided their assent to participation. The PANIC study has been carried out in accordance with the principles of the Declaration of Helsinki as revised in 2008. The trial was registered at ClinicalTrials.gov (NCT01803776). Examinations were performed in the PANIC study facilities at the University of Eastern Finland.

**Figure 1.**
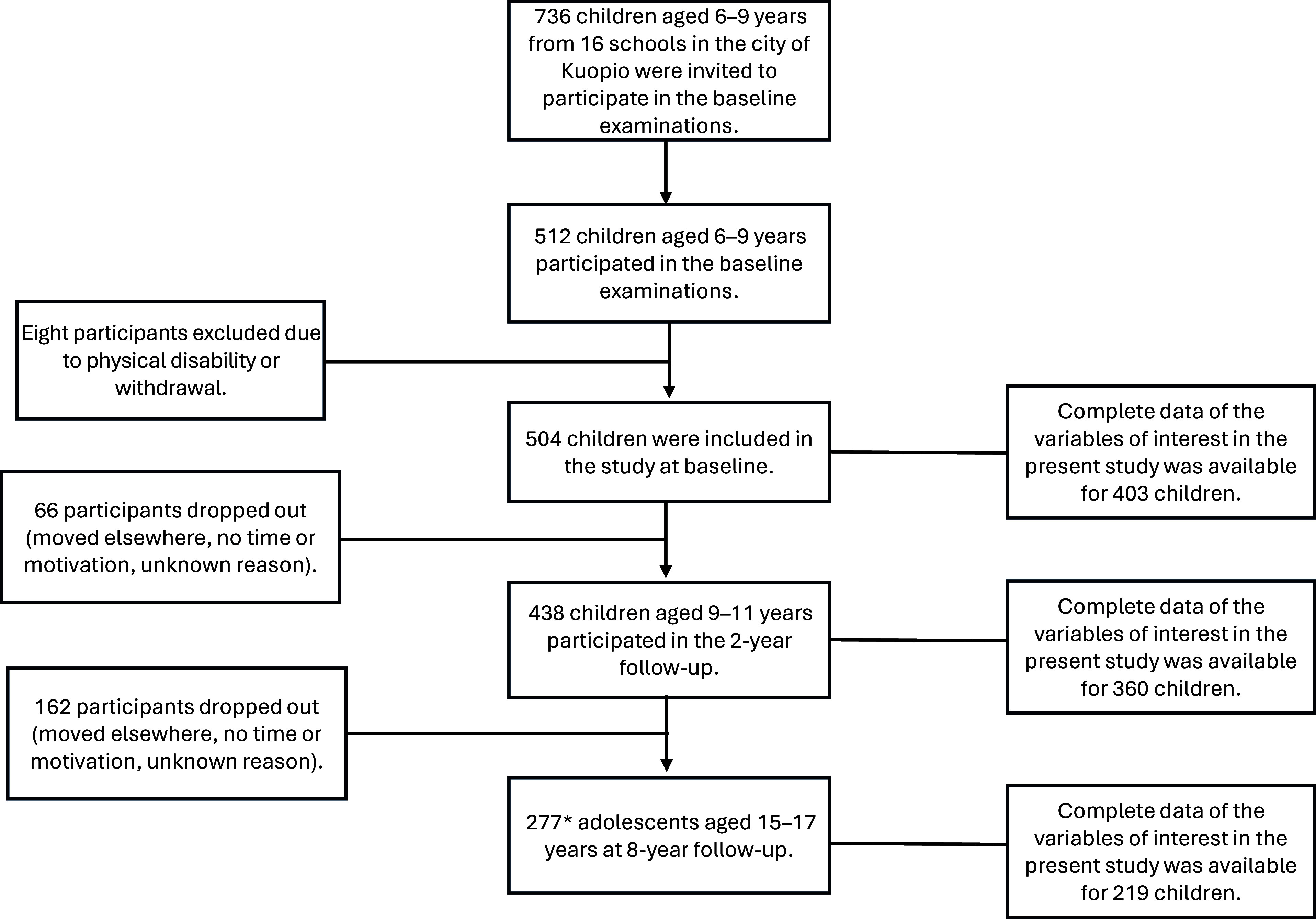
Flowchart of baseline, 2-year and 8-year examinations in the Physical Activity and Nutrition in Children (PANIC) study. *One of the participants did not attend the 2-year examinations but attended the 8-year examinations.

Altogether, 736 children aged 6–9 years were invited to participate in the baseline examinations of the PANIC study between 2007 and 2009 (Figure 1). Of all the children invited, 512 (70 %) attended. The participants did not differ in sex, age or BMI standard deviation score from all children who started the first grade in 2007–2009 based on data from the standard school health examinations performed for all Finnish children before the first grade. Eight children were excluded at baseline because of disability or withdrawal from the study. The final study sample thus included 504 children at baseline.

Overall, 440 children aged 9–11 years (87 % of those participating in baseline examinations) attended the 2-year examinations, and 277 adolescents aged 15–17 years (55 % of those participating in baseline examinations) attended the 8-year examinations. Adolescents, who participated in the 8-year examinations, did not differ from those who did not attend these examinations in terms of baseline age, BMI-SDS, sex distribution or allocation to study groups^(25)^. The present study sample consisted of 403 children at baseline, 360 children at 2 years and 219 adolescents at 8 years with complete data on variables used in the statistical analyses.

Data on the basic characteristics were available for 403 children (194 girls, 209 boys), except linoleic acid (LA), for which data from 382 children were available at baseline, and lipoprotein subclasses for which data from 401 children were available. Data for 360 children (171 girls, 189 boys) were available at 2 years and for 219 adolescents (108 females, 111 males) at 8 years, except body fat percentage, for which data for 346 children were available at 2 years and data for 217 adolescents were available at 8 years.

### Assessment of diet

At the baseline and 2-year examinations, food consumption and energy intake were assessed by food records filled out by the parents or caregivers on four predefined consecutive days, including either two weekdays and two weekend days for 99·5 % of the participants or three weekdays and one weekend day for 0·5 % of the participants^(26)^. At the 8-year examinations, food records were filled out by the adolescents themselves. The dietary data collection was conducted after blood sampling to ensure that participants had time to receive detailed guidance on how to accurately complete their dietary records. A clinical nutritionist checked the returned food records together with the children and their parents at the baseline and 2-year examinations and with the adolescents at the 8-year examinations and filled in any missing information. Food consumption (g/d) was calculated from the food records by using the Micro Nutrica^®^ dietary analysis software, version 2.5 (The Social Insurance Institution of Finland), which uses Finnish and international data on the nutrient compositions of foods^(27)^ and was regularly updated by a clinical nutritionist of the PANIC study.

We computed the Finnish Children Healthy Eating Index (FCHEI)^(28)^ to assess overall diet quality. FCHEI consists of five food categories: (1) vegetables, fruit and berries, (2) higher-fat vegetable oils and vegetable oil-based margarine (≥ 60 % fat), (3) low-fat (< 1 %) milk, (4) fish and (5) foods with high sugar content. The consumption of these foods was divided by energy intake and categorised into deciles. Deciles were scored, a higher decile getting a higher score, apart from sugary products that were inversely scored. The sum of scores from the five categories was calculated, with a minimum of 0 indicating the lowest possible diet quality and a maximum of 50 indicating the best possible diet quality.

In addition to the FCHEI food categories, we assessed separately the consumption of high-fat (≥ 1 %) milk, high-fat (≥ 1 %) sour milk products, lower-fat vegetable oils and vegetable-oil-based margarine (< 60 % fat), red meat, sausages, high-fibre (≥ 5 %) grain products and low-fibre (< 5 %) grain products. These foods also reflect diet quality and were chosen for this study based on the dietary factors used in our previous studies^(19)^ and on existing studies examining the associations of dietary factors, such as the consumption of low-fat or high-fat dairy products^(17)^, meat^(29)^ and dietary fatty acids^(30,31)^, with blood metabolites in children.

### Biochemical analyses

Venous blood samples were collected after a 12-hour fast, centrifuged and stored at a temperature of −75°C until biochemical analyses. The blood samples were collected at baseline, 2 years and 8 years. Serum concentrations of total fatty acids (FA), SFA, MUFA, PUFA, *n*-3-FA, *n*-6-FA, DHA, LA, alanine, glutamine, glycine, histidine, isoleucine, leucine, valine, total BCAA (including isoleucine, leucine and valine), aromatic amino acids (AAA, including phenylalanine and tyrosine), apoB and apoA1 as well as the sizes of VLDL, LDL and HDL particles were measured using high-throughput NMR spectroscopy metabolomics analysis (Nightingale Health Ltd, Kuopio Finland)^(32)^. We also assessed the degree of serum FA unsaturation (presence and amount of double bonds in carbon chains of FA), the percentage of SFA from total FA (SFA%), the percentage of MUFA from total FA (MUFA%), the percentage of PUFAs from total FA (PUFA%), the percentage of *n*-3-FA from total FA (*n*-3-FA%), the percentage of *n*-6-FA from total FA (*n*-6-FA%), the ratio of *n*-6-FA to *n*-3-FA (*n*-6-FA/*n*-3-FA), the percentage of DHA from total FA (DHA%), the percentage of LA from total FAs (LA%) and the ratio of apoB to apoA1 (apoB/apoA1).

### Other assessments

All the following assessments were done at baseline, 2 years and 8 years. Body weight was measured after a 12-hour fast, emptied bladder and standing in light underwear using a weight scale integrated into a calibrated InBody^®^ 720 bioelectrical impedance device (Biospace, Seoul, South Korea) to an accuracy of 0·1 kg. Body weight was measured twice, and the mean of these values was used. Body height was measured standing in the Frankfurt plane without shoes using a wall-mounted stadiometer to an accuracy of 0·1 cm. Height was measured three times, and the mean of the nearest two values was used. BMI was calculated by dividing weight (kg) by height (m) squared and BMI standard deviation score (BMI-SDS) was calculated. Body fat percentage was assessed by a dual-energy X-ray absorptiometry method with a Lunar^®^ Dual-energy X-ray absorptiometry device (GE Medical Systems, Madison, WI, USA). A research physician assessed pubertal status according to breast development for girls (scored M 1–5) and testicular volume measured by an orchidometer for boys (scored G 1–5) using the staging method described by Tanner^(33,34)^.

Physical activity was objectively measured using a combined heart rate and movement sensor (Actiheart®, CamNtech Ltd., Papworth, UK), attached to the chest with two standard ECG electrodes. Participants were instructed to wear the device continuously for at least 4 days (2 weekdays, 2 weekend days), including sleep and water-based activities, without altering their usual behavior. Heart rate data were individually calibrated using results from a maximal cycle ergometer exercise test. Moderate-to-vigorous physical activity was defined as activity exceeding an intensity of four metabolic equivalents.

### Statistical methods

Basic characteristics of the children at baseline were analysed using the SPSS Statistics software, version 27.0 (IBM Corporation, IBM SPSS Statistics for Windows, Armonk, NY, USA). The associations of changes in the indicators of diet quality with changes in serum metabolite concentrations over 8 years were analysed by linear mixed-effects models using the R Statistical Software, version 4.1.3 (2022). These models were adjusted for sex, age, body fat percentage, pubertal status and moderate-to-vigorous physical activity. To assess the sensitivity of our findings to the choice of random effects structure, we compared models with random intercepts only to models with random slopes and intercepts. A random subject-specific intercept was used in the models. The skewness of the distributions of the outcome variables was tested. For variables with skewed distributions, including DHA, DHA%, LA%, *n*-6-FA/*n*-3-FA, total BCAA, isoleucine and leucine, logarithmic transformations were made, and the linear mixed-effect analyses were conducted with the logarithmic values. Associations with *P*-values < 0·05 were considered statistically significant. A Benjamini–Hochberg false discovery rate corrected *P*-value < 0·2 was used to control for possible false positive results caused by multiple comparisons. The original sample size calculations of the PANIC study were based on the primary outcomes of the intervention, specifically fasting insulin and homeostatic model assessment of insulin resistance in children, as described earlier^(19,35)^. In short, to estimate the required sample size, we aimed to detect a difference of at least 0·30 standard deviations between the intervention group (60 % of participants) and the control group (40 %), with 80 % power and a significance level of 0·05 (two-sided test). Accounting for a potential 20 % loss due to dropouts or missing data, we determined that the study would need at least 275 children in the intervention group and 183 in the control group.

## Results

### Basic characteristics, diet quality and serum metabolites

At baseline, boys were taller, had lower body fat percentage and higher energy intake, had higher PUFA%, *n*-6-FA%, LA%, apoA1, lower total FA, SFA, MUFA, MUFA%, glutamine, apoB and apoB/apoA1 and consumed more red meat and sausages than girls (Tables 1 and 2). At baseline, there were no differences in average diameter of lipoprotein sizes but boys had higher concentrations of medium LDL and HDL particles and lower concentrations of medium and small VLDL particles and large and small LDL particles. At 2 years, boys had lower body fat percentage and higher energy intake and consumed more high-fat milk and red meat than girls. Boys also had lower alanine, glutamine and tyrosine levels, smaller LDL particles and a higher concentration of medium and small HDL particles compared with girls. At 8 years, boys were taller, weighed more and had a lower body fat percentage and higher energy intake. Their diet quality was lower. They consumed less vegetables, fruit and berries as well as high-fat sour milk products and consumed more low-fat milk, red meat, sausages and sugary products compared with girls. Boys also had lower concentrations of total FA, SFA, PUFA, *n*-3-FA, *n*-6-FA, DHA, LA and *n*-6%, DHA%, LA% and a lower degree of FA unsaturation. Boys had smaller HDL particle size, lower concentrations of large and medium HDL particles and lower serum apoA1 concentrations. Conversely, boys had higher MUFA%, *n*-6/*n*-3 ratio, glutamine, BCAA (including isoleucine, leucine and valine separately), phenylalanine and tyrosine concentrations. They also had larger VLDL particle sizes, higher concentrations of large VLDL particles and higher apoB/apoA1 ratio.

**Table 1.** Basic characteristics and dietary factors of the study participants at baseline, the 2-year and the 8-year examinations (Mean values and standard deviations; median values and interquartile ranges)

	Baseline					2 years					8 years				
	Girls (194)		Boys (209)			Girls (171)		Boys (189)			Girls (108)		Boys (111)		
	Mean/median*	sd/IQR	Mean/median*	sd/IQR	*P*-value†	Mean/median*	sd/IQR	Mean/median*	sd/IQR	*P*-value†	Mean/median*	sd/IQR	Mean/median*	sd/IQR	*P*-value†
Age (years)	7·62	0·39	7·64	0·38	0·67	9·7	0·44	9·8	0·44	0·42	15·8	0·38	15·9	0·45	0·14
Height (cm)	128	5·7	130	5·3	**0·007**	140	6·6	141	6·3	0·09	166	5·9	176	7·4	**< 0·001**
Weight (cm)	26·7	5·2	27·0	4·5	0·50	33·8	7·4	35·1	7·6	0·10	58·0	8·9	65·9	15·8	**< 0·001**
BMI (kg/m^2^)	16·2	2·2	16·0	1·9	0·49	17·1	2·6	17·5	2·8	0·19	21·1	2·8	21·1	4·2	0·99
BMI SDS	–0·2	1·1	–0·2	1·1	0·47	–0·13	1·0	–0·09	1·1	0·74	0·08	0·85	–0·11	1·1	0·16
Body fat percentage‡	22·5	7·7	16·9	7·5	**< 0·001**	25·4	8·5	21·8	9·9	**< 0·001**	28·9	6·8	17·1	9·2	**< 0·001**
Energy intake (kcal/d)	1550	284	1724	306	**< 0·001**	1577	296	1806	352	**< 0·001**	1617	436	2054	558	**< 0·001**
Finnish Children Healthy Eating Index score	23·6	6·5	22·9	7·2	0·27	24·5	7·3	23·8	7·5	0·38	24·0	7·0	22·1	7·1	**0·04**
Vegetables, fruit and berries (g/d)	216	110	206	118	0·10	205	111	210	119	0·73	270	154	192	132	**< 0·001**
Higher-fat vegetable oils and vegetable oil-based margarine (≥ 60 % fat)	10·7	8·6	11·6	9·6	0·64	13·7*	17·1	14·7*	23·6	0·32	11·8*	13·1	13·2*	18·4	0·5
Lower-fat vegetable oils and vegetable oil-based margarine (< 60 % fat)	0*	5·0	0*	5·9	0·18	0*	0	0*	0	0·68	0*	0	0*	0	0·34
Low-fat (< 1 %) milk (g/d)	350*	492	426*	544	0·08	400*	469	449*	446	0·2	200*	425	375*	529	**< 0·001**
High-fat (≥ 1 %) milk (g/d)	92·4*	227	99·6*	250	0·55	44·5*	123	74·1*	161	**0·03**	19·7*	83·5	30·9*	103	0·06
High-fat (≥ 1 %) sour milk products (g/d)	70·4*	88·4	62·5*	112·5	0·43	50*	100	50·0*	125·0	0·50	10·6*	75·0	0	100	**0·02**
Fish (g/d)	4·9	22·0	8·23*	28·1	0·13	11*	28	7·5*	28·8	0·82	5·0*	28·5	15·1*	38·4	0·17
Red meat (g/d)	51·3	26·3	60·8	33·3	**0·007**	53·4	31·9	68·6	40·3	**< 0·001**	39·8*	49·1	81·9*	80·8	**< 0·001**
Sausages (g/d)	12·1*	26·9	18·0*	26·2	**0·001**	11·8*	30·8	16·3*	33·1	0·22	1·63*	17·4	20·0*	55·0	**< 0·001**
High-fibre (≥ 5 %) grain products (g/d)	60·9	36·4	65·6	42·0	0·34	70·6	43·6	79·4	46·9	0·07	71·4*	69·9	78·1*	95·5	0·23
Low-fibre (< 5 %) grain products (g/d)	108	45·4	119	59·7	0·10	99·6	53·0	114	77·7	0·05	112	76·9	124	63·8	0·22
Sugary products (g/d)	176	129	203	144	0·08	146*	174	189*	192	0·16	104*	158	158*	275	**0·003**

* For variables with skewed distributions, medians and interquartile ranges are presented.

† Statistically significant differences between girls and boys are indicated by bolded P-values.

‡ For body fat percentage at 2 years, data from 346 children and at 8 years, data from 217 adolescents were available.

**Table 2.** Serum metabolites of the study participants at baseline, at the 2-year examinations and at the 8-year examinations (Mean values and standard deviations)

	Baseline					2 years					8 years				
	Girls (194)		Boys (209)			Girls (171)		Boys (189)			Girls (108)		Boys (111)		
	Mean	sd	Mean	sd	*P*-value†	Mean	sd	Mean	sd	*P*-value†	Mean	sd	Mean	sd	*P*-value†
Fatty acids															
Total FA (mmol/l)	11·3	1·14	11·1	1·08	**0·04**	10·2	1·30	10·3	1·36	0·47	10·0	1·54	9·44	1·36	**0·003**
SFA (mmol/l)	3·79	0·41	3·71	0·41	**0·04**	3·37	0·47	3·41	0·50	0·40	3·31	0·53	3·12	0·49	**0·006**
MUFA (mmol/l)	2·67	0·38	2·57	0·37	**0·007**	2·29	0·39	2·31	0·43	0·73	2·32	0·46	2·23	0·49	0·21
PUFA (mmol/l)	4·84	0·47	4·80	0·43	0·34	4·55	0·51	4·59	0·52	0·42	4·40	0·62	4·08	0·46	**< 0·001**
* n*-3-FA (mmol/l)	0·43	0·11	0·423	0·11	0·79	0·38	0·11	0·38	0·11	0·90	0·35	0·13	0·30	0·09	**< 0·001**
* n*-6-FA (mmol/l)	4·41	0·39	4·37	0·36	0·28	4·17	0·44	4·22	0·45	0·37	4·04	0·52	3·78	0·40	**< 0·001**
DHA (mmol/l)	0·37	0·19	0·35	0·15	0·18	0·23	0·04	0·23	0·04	0·70	0·22	0·05	0·19	0·03	**< 0·001**
LA (mmol/l)*	3·50	0·52	3·50	0·45	0·96	3·54	0·44	3·56	0·45	0·64	3·36	0·50	3·10	0·41	**< 0·001**
Degree of unsaturation	1·25	0·08	1·25	0·08	0·49	1·32	0·05	1·33	0·05	0·87	1·31	0·05	1·28	0·05	**< 0·001**
SFA/total FA (%)	33·6	1·10	33·5	1·15	0·35	33·0	1·14	33·04	1·04	0·42	33·1	1·20	33·1	1·38	0·92
MUFA/total FA (%)	23·6	1·52	23·1	1·52	**0·008**	22·4	1·46	22·3	1·76	0·56	22·99	1·52	23·48	1·96	**0·04**
PUFA/total FA (%)	42·9	2·13	43·4	2·25	**0·02**	44·67	1·78	44·68	2·10	0·97	43·9	1·91	43·4	2·46	0·09
* n*-3-FA/total FA (%)	3·75	0·79	3·81	0·83	0·45	3·64	0·86	3·63	0·86	0·97	3·46	0·83	3·11	0·73	0·60
* n*-6-FA/total FA (%)	39·1	1·87	39·6	1·96	**0·02**	41·02	1·78	41·05	2·11	0·88	40·48	1·87	40·33	2·42	**0·003**
* n*-6-FA/*n*-3-FA						11·9	3·07	12·03	3·28	0·70	12·38	3·15	13·66	3·27	**0·003**
DHA/total FA (%)	1·63	0·12	1·37	0·09	0·35	2·23	0·33	2·23	0·33	0·86	2·15	0·32	1·99	0·33	**< 0·001**
LA/total FA (%)	30·9	3·43	31·6	2·52	**0·03**	34·70	1·90	34·59	2·04	0·60	33·58	2·03	33·0	2·26	**0·04**
Amino acids															
Alanine (µmol/l)	293	53·2	285	58·7	0·15	305	57·8	292	60·6	**0·04**	364	69·3	357	67·0	0·45
Glutamine (µmol/l)	686	63·8	672	58·2	**0·02**	707	52·9	683	57·2	**< 0·001**	694	74·2	728	62·7	**< 0·001**
Glycine (µmol/l)	267	40·2	270	49·0	0·54	279	51·5	277	58·6	0·77	288	59·4	282	44·9	0·40
Histidine (µmol/l)	92·2	7·89	91·2	7·48	0·20	91·3	7·39	90·7	8·05	0·41	95·2	9·74	96·0	9·51	0·55
Total BCAA (µmol/l)	391	54·8	385	55·6	0·27	387	54·0	383	50·8	0·42	404	57·4	477	96·7	**< 0·001**
Isoleucine (µmol/l)	52·1	11·1	50·7	9·74	0·17	50·0	8·86	49·0	8·98	0·33	55·5	10·2	70·0	20·0	**< 0·001**
Leucine (µmol/l)	111	17·0	110	17·4	0·43	112	16·7	112	16·1	0·87	119	17·5	146	36·5	**< 0·001**
Valine (µmol/l)	227	30·2	224	31·0	0·27	225	31·0	222	29·3	0·30	229	33·1	263	43·4	**< 0·001**
Phenylalanine (µmol/l)	65·2	6·85	64·2	7·05	0·34	63·0	7·64	62·0	6·77	0·21	68·7	9·1	71·8	10·2	**0·02**
Tyrosine (µmol/l)	63·4	9·33	61·7	10·2	0·09	63·6	9·48	60·9	11·6	**0·007**	65·6	10·1	67·6	15·2	0·25
Average diameter of lipoprotein particles															
VLDL (nm)	37·5	0·89	37·4	0·90	0·18	37·5	0·82	37·6	0·99	0·26	37·8	0·84	38·3	1·1	**< 0·001**
LDL (nm)	23·7	0·06	23·7	0·06	0·57	23·9	0·07	23·9	0·07	**0·04**	23·9	0·07	23·9	0·06	0·23
HDL (nm)	9·73	0·15	9·75	0·13	0·22	9·71	0·16	9·70	0·16	0·66	9·74	0·15	9·60	0·15	**< 0·001**
Lipoprotein subclasses‡															
Large VLDL	6·55 × 10^−6^	4·22 × 10^−6^	5·93 × 10^−6^	3·70 × 10^−6^	0·11	4·91 × 10^−6^	2·72 × 10^−6^	5·03 × 10^−6^	3·23 × 10^−6^	0·71	5·80 × 10^−6^	2·63 × 10^−6^	6·82 × 10^−6^	3·56 × 10^−6^	**0·02**
Medium VLDL	3·40 × 10^−5^	9·80 × 10^−6^	3·12 × 10^−5^	8·85 × 10^−6^	**< 0·001**	2·32 × 10^−5^	7·59 × 10^−6^	2·31 × 10^−5^	7·71 × 10^−6^	0·86	2·31 × 10^−5^	7·88 × 10^−6^	24·3 × 10^−5^	7·60 × 10^−6^	0·27
Small VLDL	3·60 × 10^−5^	1·07 × 10^−5^	3·32 × 10^−5^	9·49 × 10^−6^	**0·006**	2·68 × 10^−5^	8·18 × 10^−6^	2·62 × 10^−5^	8·64 × 10^−6^	0·55	2·82 × 10^−5^	8·44 × 10^−6^	2·89 × 10^−5^	7·78 × 10^−6^	0·50
Large LDL	6·43 × 10^−4^	1·32 × 10^−4^	6·07 × 10^−4^	1·16 × 10^−4^	**0·003**	6·45 × 10^−4^	1·25 × 10^−4^	6·34 × 10^−4^	1·20 × 10^−4^	0·40	6·0 × 10^−4^	1·31 × 10^−4^	6·07 × 10^−4^	1·13 × 10^−4^	0·85
Medium LDL	6·06 × 10^−3^	9·47 × 10^−4^	6·31 × 10^−3^	9·12 × 10^−4^	**0·003**	2·41 × 10^−4^	4·86 × 10^−5^	2·43 × 10^−4^	4·77 × 10^−5^	0·72	2·25 × 10^−4^	4·97 × 10^−5^	2·34 × 10^−4^	5·17 × 10^−5^	0·16
Small LDL	1·93 × 10^−4^	3·04 × 10^−5^	1·83 × 10^−4^	2·66 × 10^−5^	**< 0·001**	1·48 × 10^−4^	2·43 × 10^−5^	1·47 × 10^−4^	2·24 × 10^−5^	0·79	1·37 × 10^−4^	2·43 × 10^−5^	1·39 × 10^−4^	2·28 × 10^−5^	0·57
Large HDL	2·66 × 10^−3^	9·07 × 10^−4^	2·76 × 10^−3^	7·81 × 10^−4^	0·23	1·74 × 10^−3^	6·02 × 10^−4^	1·77 × 10^−3^	6·68 × 10^−4^	0·56	1·79 × 10^−3^	5·58 × 10^−4^	1·24 × 10^−3^	4·60 × 10^−4^	**< 0·001**
Medium HDL	6·06 × 10^−3^	9·47 × 10^−4^	6·31 × 10^−3^	9·12 × 10^−4^	**0·007**	4·16 × 10^−3^	6·22 × 10^−4^	4·37 × 10^−3^	7·36 × 10^−4^	**0·004**	4·14 × 10^−3^	6·61 × 10^−4^	3·54 × 10^−3^	5·25 × 10^−4^	**< 0·001**
Small HDL	1·46 × 10^−2^	1·29 × 10^−3^	1·48 × 10^−2^	1·37 × 10^−3^	0·18	9·98 × 10^−3^	9·12 × 10^−4^	10·4 × 10^−2^	9·61 × 10^−4^	**< 0·001**	9·65 × 10^−3^	9·77 × 10^−4^	9·49 × 10^−3^	8·38 × 10^−4^	0·20
Apolipoproteins															
apo B (g/l)	0·81	0·16	0·76	0·14	**0·002**	0·71	0·13	0·70	0·13	0·55	0·67	0·14	0·67	0·12	0·98
apo A1 (g/l)	1·45	0·19	1·50	0·18	**0·02**	1·53	0·17	1·58	0·20	**< 0·01**	1·52	0·18	1·36	0·14	**< 0·001**
apo B/apo A1	0·56	0·13	0·51	0·10	**< 0·001**	0·47	0·10	0·45	0·10	0·08	0·45	0·10	0·50	0·11	**< 0·001**

BCAA, branched-chain amino acids; FA, fatty acids; LA, linoleic acid.

* For linoleic acid data from 382 children were available at baseline.

† Statistically significant differences between girls and boys are indicated by bolded P-values.

‡ For lipoprotein subclasses, data from 401 children were available at baseline.

### Associations of changes in diet quality with changes in serum fatty acids over 8 years

Increased FCHEI was associated with increased *n*-3-FA, degree of FA unsaturation, PUFA%, *n*-3-FA%, *n*-6-FA% and LA% over 8 years after adjustment for sex, age, body fat percentage, pubertal status and moderate-to-vigorous physical activity (Tables 3 and 4). Increased FCHEI was associated with decreased concentrations of SFA, MUFA, SFA%, MUFA% and *n*-6-FA/*n*-3-FA. Increased consumption of vegetables, fruit and berries was associated with increased PUFA, *n*-3-FA, *n*-3-FA% and degree of FA unsaturation but with decreased SFA% and *n*-6-FA/*n*-3-FA. Increased consumption of higher-fat vegetable oils and vegetable-oil-based margarine (≥ 60 % fat) was associated with an increased degree of FA unsaturation, PUFA%, *n*-6-FA% and decreased SFA%. Increased consumption of lower-fat vegetable oil-based margarine (< 60 % fat) was associated with increased MUFA% and decreased *n*-6-FA%. Increased consumption of low-fat milk was associated with a higher degree of FA unsaturation. A higher consumption of high-fat (≥ 1 %) milk was associated with increased SFA, SFA% and decreased PUFA% and *n*-6-FA%. Increased consumption of fish was associated with increased *n*-3-FA and *n*-3-FA% but with decreased *n*-6-FA/*n*-3-FA. Increased red meat consumption was associated with a higher degree of FA unsaturation. Increased sausage consumption was related to increased SFA% and *n*-6-FA/*n*-3-FA and decreased PUFA% and *n*-3-FA%. Increased consumption of high-fibre grain products was related to increased degree of FA unsaturation, PUFA%, *n*-3-FA%, and *n*-6-FA% but decreased SFA%, MUFA% and *n*-6-FA/*n*-3-FA. Increased consumption of sugary products was associated with increased SFA%, MUFA% and *n*-6-FA/*n*-3-FA and decreased *n*-3-FA, degree of FA unsaturation, PUFA%, *n*-3-FA% and LA%. The associations of vegetable, fruit and berry consumption with SFA%, consumption of higher-fat vegetable oils and vegetable oil-based margarine (≥ 60 % fat) with a degree of FA unsaturation, low-fat milk consumption with a degree of FA unsaturation, fish consumption with *n*-3-FA and *n*-3-FA%, red meat consumption with a degree of FA unsaturation, sausage consumption with SFA%, PUFA%, *n*-3-FA% and *n*-6-FA/*n*-3-FA and sugary product consumption with *n*-6-FA/*n*-3-FA did not remain statistically significant after false discovery rate correction.

**Table 3.** Longitudinal associations of diet quality with serum fatty acids over eight years*

	Total FA (mmol/l)	SFA (mmol/l)	MUFA (mmol/l)	PUFA (mmol/l)	*n*-3-FA (mmol/l)	*n*-6-FA (mmol/l)	DHA (mmol/l)	LA (mmol/l)	Degree of FA unsaturation
Finnish Children Healthy Eating Index	–0·007 (0·27)	–0·005 **(0·02)** †	–0·0042 **(0·04)** †	0·0017 (0·48)	0·0018 **(< 0·001)** †	–0·0001 (0·94)	0·0010 (0·45)	0·0033 (0·26)	0·0011 **(0·002)** †
Vegetables, fruit and berries (g/d)	0·0006 (0·13)	0·0001 (0·45)	0·0001 (0·29)	0·0003 **(0·02)** †	0·0001 **(< 0·001)** †	0·0002 (0·09)	0·0001 (0·15)	0·0003 (0·06)	6·17 × 10^−5^ **(0·002)** †
Higher-fat vegetable oils and vegetable oil-based margarine (≥ 60 % fat)	–0·003 (0·40)	–0·0022 (0·09)	–0·0014 (0·21)	0·0004 (0·77)	–3·34 × 10^−5^ (0·92)	0·0005 (0·70)	–0·0001 (0·89)	5·08 × 10^−5^ (0·98)	**0·0004 (0·03)**
Lower-fat vegetable oils and vegetable oil-based margarine (< 60 % fat)	0·007 (0·25)	0·0026 (0·27)	0·004 (0·002)	0·0005 (0·84)	0·0007 (0·22)	–0·0002 (0·94)	–0·0004 (0·74)	0·0004 (0·89)	–0·0004 (0·17)
Low-fat (< 1 %) milk (g/d)	–2·48 × 10^−5^ (0·87)	–2·73 × 10^−5^ (0·63)	–1·53 × 10^−5^ (0·76)	1·04 × 10^−5^ (0·86)	1·24 × 10^−5^ (0·36)	–2·53 × 10^−6^ (0·96)	–1·88 × 10^−5^ (0·56)	1·40 × 10^−6^ (0·84)	1·63 × 10^−5^ **(0·05)**
High-fat (≥ 1 %) milk (g/d)	0·0002 (0·29)	0·0002 **(0·03)** †	5·28 × 10^−5^ (0·45)	6·3 × 10^−6^ (0·94)	–5·26 × 10^−6^ (0·79)	1·11 × 10^−5^ (0·88)	–1·75 × 10^−5^ (0·70)	–9·78 × 10^−5^ (0·33)	–2·02 × 10^−5^ (0·08)
High-fat (≥ 1 %) sour milk products (g/d)	–0·0004 (0·47)	–9·84 × 10^−5^ (0·64)	–9·29 × 10^−5^ (0·62)	–0·0002 (0·33)	–3·83 × 10^−5^ (0·46)	–0·0002 (0·34)	6·47 × 10^−5^ (0·60)	–0·0003 (0·21)	–2·40 × 10^−5^ (0·45)
Fish (g/d)	–0·0007 (0·67)	–0·0005 (0·16)	–0·0001 (0·82)	–0·0001 (0·85)	0·0003 **(0·04)**	–0·0005 (0·45)	0·0003 (0·53)	0·0001 (0·89)	8·10 × 10^−5^ (0·43)
Red meat (g/d)	0·0004 (0·72)	3·90 × 10^−5^ (0·93)	–0·0002 (0·65)	0·0005 (0·27)	0·0002 (0·08)	0·0003 (0·41)	0·0003 (0·23)	0·0002 (0·79)	0·0001 **(0·03)**
Sausages (g/d)	0·0013 (0·41)	0·0008 (0·18)	0·0007 (0·21)	–8·24 × 10^−5^ (0·89)	–0·0002 (0·15)	0·0001 (0·80)	–0·0002 (0·54)	–0·0006 (0·43)	–3·33 × 10^−5^ (0·70)
High-fibre (≥ 5 %) grain products (g/d)	–0·0012 (0·18)	–0·0006 (0·05)	–0·0006 (0·06)	–6·13 × 10^−5^ (0·86)	0·0001 (0·10)	–0·0002 (0·53)	4·69 × 10^−5^ (0·81)	0·0004 (0·85)	0·0001 **(0·01)** †
Low-fibre (< 5 %) grain products (g/d)	0·0002 (0·80)	0·0001 (0·70)	4·5 × 10^−5^ (0·85)	6·42 × 10^−6^ (0·98)	–8·13 × 10^−5^ (0·20)	8·53 × 10^−5^ (0·72)	–0·0001 (0·35)	–8·79 × 10^−5^ (0·80)	–5·39 × 10^−5^ (0·17)
Sugary products (g/d)	0·0002 (0·46)	0·0002 (0·16)	0·0002 (0·09)	–9·37 × 10^−5^ (0·42)	–7·79 × 10^−5^ **(0·004)** †	–1·31 × 10^−5^ (0·90)	6·22 × 10^−6^ (0·92)	–0·0003 (0·07)	–3·00 × 10^−5^ **(0·07)** †

FA, fatty acids; LA, linoleic acid.

* Data are unstandardised regression coefficients from linear mixed-effects models adjusted for sex, age, body fat percentage, pubertal status and physical activity. *P*-values are reported in parentheses. Statistically significant associations are indicated by bolded *P*-values.

† Association remained statistically significant after Benjamini–Hochberg false discovery rate correction for multiple testing.

**Table 4.** Longitudinal associations of diet quality with serum fatty acid ratios over eight years*

	SFA/total FA (%)	MUFA/total FA (%)	PUFA/total FA (%)	*n*-3-FA/total FA (%)	*n*-6-FA/total FA (%)	*n*-6-FA/*n*-3-FA	DHA/total FA (%)	LA/total FA (%)
Finnish Children Healthy Eating Index	–0·027 **(< 0·001)** †	–0·022 **(0·006)** †	0·050 **(< 0·001)** †	0·020 **(< 0·001)** †	0·030 **(0·003)** †	–0·0053 **(< 0·001)** †	0·0020 (0·1)	0·0035 **(0·009)** †
Vegetables, fruit and berries (g/d)	–0·001 **(0·02)**	–2·69 × 10^−5^ (0·96)	0·0009 (0·18)	0·0010 **(< 0·001)** †	–0·0002 (0·78)	–0·0003 **(< 0·001)** †	6·16 × 10^−5^ (0·41)	7·43 × 10^−5^ (0·36)
Higher-fat vegetable oils and vegetable oil-based margarine (≥ 60 % fat)	–0·013 **(< 0·001)** †	–0·0072 (0·13)	0·020 **(0·002)** †	0·0007 (0·76)	0·020 **(0·001)** †	–0·0001 (0·88)	0·0002 (0·74)	0·0011 (0·16)
Lower-fat vegetable oils and vegetable oil-based margarine (< 60 % fat)	–0·001 (0·84)	0·022 **(0·007)** †	–0·022 (0·06)	0·0040 (0·36)	–0·026 **(0·01)** †	–0·0009 (0·51)	–0·0012 (0·35)	–0·0006 (0·68)
Low-fat (< 1 %) milk (g/d)	–0·0002 (0·24)	–0·0001 (0·48)	0·0003 (0·23)	0·0001 (0·27)	0·0002 (0·44)	–7·87 × 10^−6^ (0·81)	3·24 × 10^−5^ (0·28)	3·18 × 10^−5^ (0·32)
High-fat (≥ 1 %) milk (g/d)	0·0009 **(< 0·001)** †	2·53 × 10^−6^ (0·99)	–0·0009 **(0·02)** †	–0·0001 (0·42)	–0·0008 **(0·03)** †	–1·54 × 10^−5^ (0·74)	–3·81 × 10^−5^ (0·37)	–3·49 × 10^−5^ (0·44)
High-fat (≥ 1 %) sour milk products (g/d)	0·0005 (0·36)	–0·0002 (0·77)	–0·0003 (0·81)	–0·0002 (0·59)	–7·06 × 10^−5^ (0·94)	6·15 × 10^−5^ (0·62)	0·0001 (0·36)	4·89 × 10^−6^ (0·97)
Fish (g/d)	–0·0029 (0·10)	0·0003 (0·92)	0·0027 (0·42)	0·0039 **(0·003)**	–0·0014 (0·65)	–0·0011 **(0·004)** †	0·0003 (0·38)	3·20 × 10^−5^ (0·94)
Red meat (g/d)	–0·001 (0·36)	–0·0025 (0·11)	0·0035 (0·09)	0·0013 (0·10)	0·0023 (0·24)	–0·0003 (0·28)	0·0003 (0·24)	–0·0001 (0·68)
Sausages (g/d)	0·003 **(0·04)**	0·0037 (0·08)	–0·0067 **(0·02)**	–0·0022 **(0·04)**	–0·046 (0·08)	0·0007 **(0·04)**	–0·0004 (0·19)	–0·0006 (0·08)
High-fibre (≥ 5 %) grain products (g/d)	–0·002 **(0·006)** †	–0·0030 **(0·01)** †	0·0054 **(< 0·001)** †	0·0018 **(0·004)** †	0·0034 **(0·02)** †	–0·0005 **(0·02)** †	0·0002 (0·21)	0·0003 (0·16)
Low-fibre (< 5 %) grain products (g/d)	0·0004 (0·51)	0·0002 (0·87)	–0·0006 (0·65)	–0·0008 (0·13)	0·0002 (0·86)	0·0002 (0·21)	–0·0002 (0·29)	–8·4 × 10–^5^ (0·60)
Sugary products (g/d)	0·0008 **(0·007)** †	0·0010 **(0·01)** †	–0·0017 **(0·001)** †	–0·0008 **(< 0·001)** †	–0·0010 (0·05)	0·0002 **(0·002)**	–3·43 × 10^−5^ (0·57)	–0·0002 **(< 0·001)** †

FA, fatty acids; LA, linoleic acid.

* Data are unstandardised regression coefficients from linear mixed-effects models adjusted for sex, age, body fat percentage, pubertal status and physical activity. *P*-values are reported in parentheses. Statistically significant associations are indicated by bolded *P*-values.

† Associations remained statistically significant after Benjamini–Hochberg false discovery rate correction for multiple testing.

### Associations of changes in diet quality with changes in serum amino acids over 8 years

Increased FCHEI was associated with decreased alanine over 8 years after adjustment for sex, age, body fat percentage, pubertal status and physical activity (Table 5). Increased consumption of vegetables, fruit, and berries was associated with decreased alanine, glycine and isoleucine. Increased consumption of lower-fat vegetable oils and vegetable-oil-based margarine (< 60 % fat) was related to increased alanine. Increased consumption of low-fat (< 1 %) milk was related to increased leucine and phenylalanine but decreased alanine. Increased consumption of high-fat (≥ 1 %) milk was related to increased total BCAA, isoleucine, leucine, valine, phenylalanine and tyrosine but decreased glycine. Increased consumption of high-fat (≥1 %) sour milk products was associated with higher total BCAA and valine. Increased red meat consumption was associated with increased histidine but decreased isoleucine. All these longitudinal associations except the association between lower-fat vegetable oils and vegetable-oil-based margarine (< 60 % fat) with alanine, low-fat (< 1 %) milk and alanine, leucine and phenylalanine, high-fat (≥ 1 %) milk with glycine, high-fat (≥ 1 %) sour milk products and total BCAA and valine and the association between red meat consumption with histidine and isoleucine remained statistically significant after false discovery rate correction.

**Table 5.** Longitudinal associations of diet quality with serum amino acids over 8 years*

	Alanine (µmol/l)	Glutamine (µmol/l)	Glycine (µmol/l)	Histidine (µmol/l)	Total BCAAs (µmol/l)	Isoleucine (µmol/l)	Leucine (µmol/l)	Valine (µmol/l)	Phenylalanine (µmol/l)	Tyrosine (µmol/l)
Finnish Children Healthy Eating Index	–0·0008 **(0·008)** †	–0·0002 (0·55)	–0·0004 (0·09)	–6·80 × 10^−5^ (0·10)	–0·0001 (0·89)	–0·0009 (0·35)	0·0002 (0·85)	–6·01 × 10^−5^ (0·72)	8·36 × 10^−6^ (0·83)	2·36 × 10^−5^ (0·64)
Vegetables, fruit and berries (g/d)	–3·96 × 10^−5^ **(0·03)** †	–2·74 × 10^−5^ (0·13)	–2·83 × 10^−5^ **(0·04)** †	–1·49 × 10^−6^ (0·54)	–6·11 × 10^−5^ (0·17)	–0·0001 **(0·03)** †	–4·32 × 10^−5^ (0·37)	–1·29 × 10^−5^ (0·20)	–5·78 × 10^−7^ (0·80)	–1·46 × 10^−6^ (0·63)
Higher-fat vegetable oils and vegetable oil-based margarine (≥ 60 % fat)	–0·0001 (0·55)	0·0001 (0·51)	0·0001 (0·33)	–5·09 × 10^−6^ (0·83)	–0·0002 (0·62)	–0·0008 (0·20)	3·55 × 10^−5^ (0·94)	–7·58 × 10^−5^ (0·44)	–1·64 × 10^−5^ (0·47)	7·81 × 10^−6^ (0·79)
Lower-fat vegetable oils and vegetable oil-based margarine (< 60 % fat)	0·0007 **(0·03)**	–0·0001 (0·71)	0·0001 (0·60)	4·28 × 10^−5^ (0·31)	–0·0002 (0·83)	–0·0001 (0·91)	–0·0004 (0·60)	–1·52 × 10^−6^ (0·99)	3·91 × 10^−5^ (0·32)	1·39 × 10^−5^ (0·79)
Low-fat (< 1 %) milk (g/d)	–1·51 × 10^−5^ **(0·04)**	–2·76 × 10^−6^ (0·71)	–6·79 × 10^−6^ (0·24)	7·64 × 10^−7^ (0·44)	2·62 × 10^−5^ (0·14)	–5·52 × 10^−6^ (0·82)	3·78 × 10^−5^ **(0·05)**	4·92 × 10^−6^ (0·22)	2·31 × 10^−6^ **(0·01)**	1·78 × 10^−6^ (0·15)
High-fat (≥ 1 %) milk (g/d)	3·59 × 10^−6^ (0·73)	–9·24 × 10^−6^ (0·38)	–2·32 × 10^−5^ **(0·004)**	1·68 × 10^−6^ (0·23)	9·45 × 10^−5^ **(< 0·001)** †	9·38 × 10^−5^ **(0·005)** †	7·64 × 10^−5^ **(0·005)** †	2·53 × 10^−5^ **(< 0·001)** †	3·18 × 10^−6^ **(0·02)** †	4·37 × 10^−6^ **(0·01)** †
High-fat (≥ 1 %) sour milk products (g/d)	–3·22 × 10^−5^ (0·26)	–1·38 × 10^−5^ (0·63)	–2·21 × 10^−5^ (0·30)	–9·89 × 10^−9^ (1·00)	0·0001 **(0·05)**	0·0001 (0·20)	0·0001 (0·13)	3·53 × 10^−5^ **(0·02)**	5·45 × 10^−6^ (0·13)	6·57 × 10^−6^ (0·17)
Fish (g/d)	–4·54 × 10^−5^ (0·62)	–3·97 × 10^−5^ (0·67)	–1·25 × 10^−5^ (0·85)	–1·40 × 10^−5^ (0·26)	–0·0002 (0·33)	–0·0002 (0·54)	–0·0003 (0·16)	–4·66 × 10^−5^ (0·36)	2·36 × 10^−6^ (0·84)	–5·08 × 10^−6^ (0·74)
Red meat (g/d)	7·58 × 10^−6^ (0·90)	5·94 × 10^−5^ (0·30)	–3·85 × 10^−5^ (0·36)	1·87 × 10^−5^ **(0·02)**	–8·73 × 10^−6^ (0·54)	–0·0004 **(0·03)**	–0·0001 (0·48)	–1·72 × 10^−7^ (1·00)	–3·73 × 10^−6^ (0·62)	5·90 × 10^−6^ (0·55)
Sausages (g/d)	8·80 × 10^−5^ (0·27)	–9·52 × 10^−6^ (0·90)	1·12 × 10^−5^ (0·85)	4·88 × 10^−6^ (0·65)	0·0002 (0·31)	0·0002 (0·44)	0·0002 (0·47)	5·48 × 10^−5^ (0·21)	8·86 × 10^−6^ (0·38)	1·30 × 10^−5^ (0·33)
High-fibre (≥ 5 %) grain products (g/d)	–3·38 × 10^−5^ (0·45)	1·42 × 10^−5^ (0·75)	–1·60 × 10^−5^ (0·62)	4·50 × 10^−6^ (0·45)	–6·99 × 10^−5^ (0·51)	–0·0002 (0·26)	–5·64 × 10^−5^ (0·63)	–1·28 × 10^−5^ (0·60)	–4·40 × 10^−6^ (0·43)	3·53 × 10^−6^ (0·63)
Low-fibre (< 5 %) grain products (g/d)	–2·28 × 10^−5^ (0·52)	5·66 × 10^−5^ (0·11)	–1·85 × 10^−5^ (0·48)	3·84 × 10^−6^ (0·42)	0·0001 (0·23)	8·58 × 10^−5^ (0·46)	0·0001 (0·17)	2·66 × 10^−5^ (0·17)	2·22 × 10^−6^ (0·62)	1·04 × 10^−5^ (0·08)
Sugary products (g/d)	1·37 × 10^−5^ (0·35)	5·56 × 10^−6^ (0·71)	1·17 × 10^−5^ (0·29)	2·17 × 10^−6^ (0·28)	–4·39 × 10^−5^ (0·22)	–1·74 × 10^−5^ (0·72)	–5·14 × 10^−5^ (0·19)	1·04 × 10^−5^ (0·20)	–2·02 × 10^−6^ (0·28)	–2·21 × 10^−6^ (0·37)

BCAA, branched chain amino acids.

* Data are unstandardised regression coefficients from a linear mixed effects model adjusted for age, sex, body fat percentage, pubertal status and physical activity. *P*-values are reported in parentheses. Statistically significant associations are indicated by bolded *P*-values.

† Associations remained statistically significant after Benjamini–Hochberg false discovery rate correction for multiple testing.

### Associations of changes in diet quality with changes in serum lipoprotein particle sizes and serum apolipoproteins over 8 years

Increased FCHEI was associated with decreased VLDL particle size and lower concentration of large VLDL particles after adjustment for sex, age, body fat percentage, pubertal status and physical activity (Tables 6 and 7). Increased consumption of vegetables, fruit and berries was associated with higher concentrations of medium and small VLDL particles and large and small LDL particles. Increased consumptions of lower-fat vegetable oils and vegetable-oil-based margarine (< 60 % fat) were associated with increased VLDL particle size and higher concentration of large VLDL particles, decreased LDL particle size and lower concentration of large HDL particles. Increased consumption of sugary products was associated with increased VLDL particle size and higher concentration of large and small VLDL particles and a smaller HDL particle size and lower concentration of large HDL particles. The associations of lower-fat vegetable oils and vegetable oil-based margarine (< 60 % fat) with VLDL particle size and lower concentrations of large HDL particles, and consumption of sugar products with the concentration of medium VLDL did not remain statistically significant after false discovery rate correction.

**Table 6.** Longitudinal associations of diet quality with lipoprotein particle size over 8 years*

	VLDL diameter (nm)	LDL diameter (nm)	HDL diameter (nm)
Finnish Healthy Eating Index	–0·012 **(0·008)** †	0·0008 (0·06)	0·0011 (0·10)
Vegetables, fruit and berries (g/d)	0·0004 (0·13)	2·01 × 10^−5^ (0·42)	–5·44 × 10^−5^ (0·18)
Higher-fat vegetable oils and vegetable oil-based margarine (≥60 % fat)	–0·0039 (0·13)	0·0002 (0·44)	0·0004 (0·31)
Lower-fat vegetable oils and vegetable oil-based margarine (< 60 % fat)	0·0098 **(0·03)**	–0·0011 **(0·009)** †	–0·0009 (0·21)
Low-fat (< 1 %) milk (g/d)	–3·82 × 10^−5^ (0·73)	5·83 × 10^−6^ (0·56)	–1·34 × 10^−6^ (0·94)
High-fat (≥ 1 %) milk (g/d)	1·25 × 10^−5^ (0·94)	6·32 × 10^−6^ (0·66)	–1·80 × 10^−5^ (0·45)
High-fat (≥ 1 %) sour milk products (g/d)	–0·0004 (0·39)	3·46 × 10^−6^ (0·93)	3·37 × 10^−5^ (0·59)
Fish (g/d)	–0·0003 (0·81)	0·0001 (0·42)	3·44 × 10^−5^ (0·86)
Red meat (g/d)	0·0007 (0·40)	4·95 × 10^−5^ (0·53)	–0·0002 (0·14)
Sausages (g/d)	0·0021 (0·07)	2·58 × 10^−5^ (0·81)	4·53 × 10^−5^ (0·79)
High-fibre (≥ 5 %) grain products (g/d)	–0·0009 (0·17)	9·46 × 10^−6^ (0·88)	6·82 × 10^−5^ (0·48)
Low-fibre (< 5 %) grain products (g/d)	0·0009 (0·09)	–6·02 × 10^−5^ (0·21)	–3·72 × 10^−5^ (0·63)
Sugary products (g/d)	0·0006 **(0·001)** †	–3·66 × 10^−6^ (0·07)	–7·10 × 10^−5^ **(0·03)** †

* Data are unstandardised regression coefficients from linear mixed-effects models adjusted for sex, age, body fat percentage, pubertal status and physical activity. *P*-values are reported in parentheses. Statistically significant associations are indicated by bolded *P*-values.

† Associations remained statistically significant after Benjamini–Hochberg false discovery rate correction for multiple testing.

**Table 7.** Longitudinal associations of diet quality and dietary factors with lipoprotein subclasses over 8 years*

	Large VLDL (mmol/l)	Medium VLDL (mmol/l)	Small VLDL (mmol/l)	Large LDL (mmol/l)	Medium LDL (mmol/l)	Small LDL (mmol/l)	Large HDL (mmol/l)	Medium HDL (mmol/l)	Small HDL (mmol/l)
Finnish Children Healthy Eating Index	–4·58 × 10^−8^ **(0·01)** †	–6·31 × 10^−8^ (0·13)	–7·43 × 10^−8^ (0·11)	–1·82 × 10^−7^ (0·75)	–4·11 × 10^−7^ (0·11)	–1·16 × 10^−7^ (0·35)	3·88 × 10^−6^ (0·26)	–3·78 × 10^−6^ (0·37)	–1·16 × 10^−7^ (0·35)
Vegetables, fruit and berries (g/d)	1·68 × 10^−9^ (0·12)	5·70 × 10^−9^ **(0·02)** †	6·50 × 10^−9^ **(0·02)** †	6·78 × 10^−8^ **(0·05)** †	2·86 × 10^−8^ (0·06)	1·67 × 10^−8^ **(0·02)** †	–2·23 × 10^−7^ (0·27)	–2·00 × 10^−7^ (0·42)	1·98 × 10^−7^ (0·63)
Higher-fat vegetable oils and vegetable oil-based margarine (≥ 60 % fat)	–1·48 × 10^−8^ (0·16)	–2·28 × 10^−8^ (0·34)	–2·05 × 10^−8^ (0·44)	–2·93 × 10^−7^ (0·38)	1·45 × 10^−7^ (0·32)	–6·58 × 10^−8^ (0·35)	1·45 × 10^−6^ (0·46)	8·16 × 10^−7^ (0·73)	–6·58 × 10^−8^ (0·35)
Lower-fat vegetable oils and vegetable oil-based margarine (< 60 % fat)	4·47 × 10^−8^ **(0·02)** †	–1·91 × 10^−10^ (0·85)	7·33 × 10^−8^ (0·12)	–1·04 × 10^−6^ (0·08)	–1·47 × 10^−7^ (0·58)	–1·19 × 10^−7^ (0·35)	–7·24 × 10^−6^ **(0·04)**	–5·94 × 10^−6^ (0·16)	–1·19 × 10^−7^ (0·35)
Low-fat (< 1 %) milk (g/d)	–1·28 × 10^−10^ (0·77)	–1·91 × 10^−10^ (0·85)	–3·71 × 10^−10^ (0·74)	–1·27 × 10^−9^ (0·93)	–3·27 × 10^−9^ (0·61)	–4·97 × 10^−10^ (0·87)	–6·00 × 10^−8^ (0·48)	–1·55 × 10^−7^ (0·13)	–4·97 × 10^−10^ (0·87)
High-fat (≥ 1 %) milk (g/d)	–1·98 × 10^−10^ (0·75)	1·50 × 10^−9^ (0·30)	1·35 × 10^−9^ (0·40)	3·28 × 10^−8^ (0·11)	1·42 × 10^−8^ (0·11)	6·05 × 10^−9^ (0·17)	4·56 × 10^−8^ (0·70)	2·77 × 10^−7^ (0·06)	6·05 × 10^−9^ (0·17)
High-fat (≥ 1 %) sour milk products (g/d)	–2·52 × 10^−10^ (0·88)	–1·04 × 10^−9^ (0·79)	–1·42 × 10^−9^ (0·74)	1·78 × 10^−9^ (0·97)	–6·83 × 10^−9^ (0·77)	1·38 × 10^−9^ (0·90)	–8·92 × 10^−8^ (0·78)	–3·61 × 10^−7^ (0·35)	1·38 × 10^−9^ (0·90)
Fish (g/d)	2·37 × 10^−9^ (0·67)	–1·03 × 10^−9^ (0·93)	7·50 × 10^−9^ (0·58)	–7·45 × 10^−8^ (0·66)	–8·58 × 10^−8^ (0·25)	–3·30 × 10^−8^ (0·36)	–8·68 × 10^−7^ (0·39)	–2·16 × 10^−6^ (0·08)	–3·30 × 10^−8^ (0·36)
Red meat (g/d)	1·49 × 10^−9^ (0·67)	1·01 × 10^−8^ (0·18)	1·19 × 10^−8^ (0·16)	7·66 × 10^−8^ (0·47)	4·53 × 10^−8^ (0·33)	1·40 × 10^−8^ (0·53)	–1·08 × 10^−6^ (0·08)	7·91 × 10^−7^ (0·29)	1·40 × 10^−8^ (0·53)
Sausages (g/d)	7·18 × 10^−9^ (0·13)	1·02 × 10^−8^ (0·33)	1·08 × 10^−8^ (0·36)	1·31 × 10^−7^ (0·37)	2·86 × 10^−8^ (0·66)	2·84 × 10^−8^ (0·36)	7·91 × 10^−7^ (0·36)	6·83 × 10^−7^ (0·51)	2·84 × 10^−8^ (0·36)
High-fibre (≥ 5 %) grain products (g/d)	–3·33 × 10^−9^ (0·21)	–6·05 × 10^−9^ (0·30)	–5·67 × 10^−9^ (0·39)	–9·25 × 10^−8^ (0·26)	–4·40 × 10^−8^ (0·23)	–1·67 × 10^−8^ (0·34)	–1·32 × 10^−7^ (0·78)	–5·00 × 10^−7^ (0·39)	–1·67 × 10^−8^ (0·34)
Low-fibre (< 5 %) grain products (g/d)	1·95 × 10^−9^ (0·35)	3·38 × 10^−9^ (0·47)	2·40 × 10^−9^ (0·65)	–7·00 × 10^−9^ (0·92)	1·49 × 10^−8^ (0·61)	6·80 × 10^−9^ (0·63)	–2·42 × 10^−7^ (0·53)	–5·98 × 10^−8^ (0·90)	6·80 × 10^−9^ (0·63)
Sugary products (g/d)	2·84 × 10^−9^ **(0·001)** †	4·07 × 10^−9^ **(0·04)**	5·09 × 10^−9^ **(0·02)** †	–5·38 × 10^−9^ (0·85)	1·76 × 10^−8^ (0·15)	4·61 × 10^−9^ (0·44)	–3·59 × 10^−7^ **(0·03)** †	–1·65 × 10^−7^ (0·41)	4·61 × 10^−9^ (0·44)

* Data are unstandardised regression coefficients from linear mixed-effects models adjusted for sex, age, body fat percentage, pubertal status and physical activity. *P* values are reported in parentheses. Statistically significant associations are indicated by bolded *P* values.

† Associations remained statistically significant after Benjamini–Hochberg false discovery rate correction for multiple testing.

No associations were found between diet quality and food consumption with apo over 8 years (Table 8).

**Table 8.** Longitudinal associations of diet quality and dietary factors with apolipoproteins over 8 years*

	apo B (g/l)	apo A1 (g/l)	apo B/apo A1
Finnish Children Healthy Eating Index	–0·0005 (0·40)	–0·0005 (0·61)	–0·0003 (0·50)
Vegetables, fruit and berries (g/d)	7·22 × 10^−5^ (0·06)	–4·39 × 10^−5^ (0·40)	5·61 × 10^−5^ (0·05)
Higher-fat vegetable oils and vegetable oil-based margarine (≥ 60 % fat)	–0·0004 (0·30)	0·0006 (0·27)	–0·0005 (0·09)
Lower-fat vegetable oils and vegetable oil-based margarine (< 60 % fat)	–0·0009 (0·18)	–0·0017 (0·05)	5·92 × 10^−5^ (0·91)
Low-fat (< 1 %) milk (g/d)	–3·50 × 10^−6^ (0·83)	–1·87 × 10^−5^ (0·39)	4·08 × 10^−6^ (0·74)
High-fat (≥ 1 %) milk (g/d)	3·84 × 10^−5^ (0·09)	4·50 × 10^−5^ (0·14)	1·17 × 10^−5^ (0·50)
High-fat (≥ 1 %) sour milk products (g/d)	–2·94 × 10^−6^ (0·96)	–5·05 × 10^−5^ (0·53)	5·42 × 10^−6^ (0·90)
Fish (g/d)	–9·97 × 10^−5^ (0·59)	–0·0004 (0·15)	5·22 × 10^−5^ (0·71)
Red meat (g/d)	0·0001 (0·37)	–7·62 × 10^−5^ (0·64)	0·0001 (0·23)
Sausages (g/d)	0·0002 (0·34)	0·0001 (0·59)	8·41 × 10^−5^ (0·49)
High-fibre (≥ 5 %) grain products (g/d)	–9·31 × 10^−5^ (0·30)	–8·57 × 10^−5^ (0·49)	–4·36 × 10^−5^ (0·52)
Low-fibre (< 5 %) grain products (g/d)	2·93 × 10^−6^ (0·97)	1·24 × 10^−6^ (0·99)	4·10 × 10^−6^ (0·94)
Sugary products (g/d)	1·03 × 10^−5^ (0·74)	–4·51 × 10^−5^ (0·28)	2·71 × 10^−5^ (0·24)

* Data are unstandardised regression coefficients from linear mixed-effects models adjusted for sex, age, body fat percentage, pubertal status and physical activity. *P*-values are reported in parentheses. Statistically significant associations are indicated by bolded *P*-values.

## Discussion

We observed that improved diet quality, as indicated by increased consumptions of vegetables, fruit and berries, higher-fat vegetable oils and vegetable oil-based margarine (≥ 60 % fat), fish and high-fibre grain products and a decreased consumption of sugary products were longitudinally associated with increased serum PUFA and decreased serum MUFA and SFA from childhood to adolescence. Increased consumptions of dairy products and sausages were also longitudinally related to increased serum BCAA and AAA. Improved overall diet quality was related to decreased serum VLDL particle size and lower concentration of large VLDL particles. Conversely, worsened diet quality, as indicated by increased consumption of lower-fat vegetable oils and vegetable oil-based margarines (< 60 % fat) and sugary products, was longitudinally associated with increased VLDL size. Additionally, increased consumption of lower-fat vegetable oils and vegetable oil-based margarines (< 60 % fat) were associated with decreased LDL size.

### Diet quality and serum fatty acids

Increased FCHEI was related to increased serum PUFA and decreased serum SFA and MUFA from childhood to adolescence. These results align with the findings of our previous cross-sectional study, in which better diet quality was related to higher serum PUFA measured with NMR, and our 2-year intervention study, in which the intervention group showed an increased plasma proportion of PUFA in cholesteryl esters and phospholipids measured with gas chromatography^(19,36)^. Also supporting our observations, another Finnish intervention study showed that better adherence to a dietary intervention based on nutritional recommendations was associated with higher serum PUFA and lower serum SFA from childhood to adulthood^(37)^. In this study, increased consumption of vegetables, fruit and berries and high-fibre grain products were associated with increased serum PUFA, such as *n*-3-FA and decreased serum proportion of SFA to total FA from childhood to adolescence. A higher consumption of vegetables, fruit and berries might be an indicator of better overall diet quality, which then is reflected in a more favourable serum fatty acid profile. It is also possible that the participants who ate more vegetables, fruit and berries were more health conscious and thus ate more foods containing lots of PUFA and had an improved serum fatty acid profile than other participants. Additionally, high-fibre grain products are important sources of PUFA in our study population^(26)^. We observed that an increased consumption of direct dietary sources of PUFA, such as higher-fat vegetable oils and vegetable-oil-based margarine (≥ 60 % fat) and fish, was reflected as an increased serum PUFA%, serum degree of FA unsaturation and decreased serum SFA%. Similar results from cross-sectional studies in children have been described earlier^(31,38)^. In this study, an increased sugary product consumption was reflected as decreased serum PUFA and increased serum SFA. This might be related to the increased intake of SFA since sugary products such as ice cream and chocolate are major dietary sources of SFA in our study population of children^(26)^.

Higher circulating levels of PUFA have been associated with a lower risk of many cardiometabolic disturbances, such as insulin resistance and elevated blood pressure, and are thought to be beneficial for cardiometabolic health due to their anti-inflammatory properties in adults^(10,39,40)^. Moreover, blood *n*-3-FA have been inversely associated with cardiometabolic risk factors, such as elevated blood pressure, since childhood^(41)^. Thus, our results suggest that improved diet quality, characterised by an increased consumption of unsaturated fat products and fibre as well as a reduced consumption of sugary products, has beneficial effects on FA metabolism from childhood to adolescence.

### Diet quality and serum amino acids

Increased FCHEI was associated with decreased serum alanine from childhood to adolescence, the results being consistent with our previous cross-sectional findings^(19)^. Interestingly, in the present longitudinal study, some indicators of impaired diet quality were associated with increased serum BCAA, associations which were not seen in our earlier cross-sectional study. Increased consumptions of low-fat and high-fat milk were reflected in increased serum BCAA and AAA, whereas an increased consumption of vegetables, fruit and berries was associated with decreased serum BCAA and AAA. Our longitudinal findings are similar to those of a Danish intervention study in children^(29)^, in which an increased protein intake as meat or dairy was reflected in increased serum BCAA in the intervention and control groups. In another intervention study among infants, complementary feeding of a protein-rich diet with meat or dairy products increased serum BCAA levels^(42)^. Moreover, in a Finnish dietary intervention study aiming towards dietary choices based on nutritional recommendations, serum BCAA levels were lower among boys in the intervention group than among boys in the control group, although the difference was statistically nonsignificant^(23)^. These observations together suggest that diet quality, particularly diets high in protein, predominantly derived from dairy and meat sources, and a lower consumption of vegetables, fruit and berries affect amino acid metabolism and may lead to higher blood concentrations of BCAA and AAA. Our results also indicated that these associations are independent of physical activity. These findings may be clinically important as higher circulating BCAA has been associated with a higher risk of insulin resistance in children, adolescents, and adults^(9,11,43)^. Higher circulating levels of some AAA, such as phenylalanine, have also been linked to adverse cardiovascular outcomes in adults^(9,44)^ and insulin resistance in children^(11)^.

### Diet quality and serum lipoprotein particle sizes

Improved overall diet quality was associated with decreased serum VLDL particle size from childhood to adolescence, whereas impaired diet quality and increased consumption of lower-fat vegetable oils and vegetable oil-based margarine (< 60 % fat) and sugary products were associated with increased serum VLDL particle size and higher concentration of large VLDL particles. These longitudinal findings are consistent with the results of our previous cross-sectional study^(19)^. However, in dietary intervention studies, no such effect of diet affecting circulating VLDL particle size in children has been observed^(37,45)^. Nevertheless, in adults, better diet quality has been associated with smaller circulating VLDL particle size^(46)^, thus supporting our present results. A plausible mechanism for the observed associations of an increased consumption of high-fat and high-sugar foods with increased VLDL particle size could be increased liver adiposity due to the increased dietary intake of SFA and fructose^(19)^. Liver adiposity has been linked to the secretion of larger VLDL particles^(47)^, which in turn are associated with the formation of smaller, more dense LDL particles that are atherogenic due to their fibrinolytic, oxidative and inflammatory properties^(48)^. In fact, smaller LDL particle size has been associated with adverse cardiovascular health outcomes, such as coronary artery disease and myocardial infarction, in adults^(49,50)^. Therefore, larger VLDL particles might contribute to the development of CVD which underlines the biological significance of diet quality and the possible public health relevance of our findings.

In this study, increased consumption of sugary products was associated with decreased serum HDL particle size and a lower concentration of large HDL particles from childhood to adolescence. Some studies have observed that smaller circulating HDL particles are associated with a higher risk of CVD, such as CHD, in adults^(51)^. This is possibly due to the higher susceptibility of smaller HDL particles to degradation compared to larger HDL particles^(52,53)^. We also observed that an increased consumption of lower-fat vegetable oils and vegetable oil-based margarine (< 60 % fat) was associated with decreased serum LDL particle size, which is unfavourable for cardiovascular health due to the atherogenic nature of small LDL particles^(48–50)^. In adults, better diet quality has been associated with a lower quantity of circulating small LDL and HDL particles^(46,54)^, while studies examining the associations of dietary factors with lipoprotein particle sizes in children are scarce. Of single dietary factors, a higher PUFA intake has been associated with larger circulating HDL and LDL particle sizes in adults^(55,56)^. Thus, the present findings concerning the associations of increased consumption of sugary products and lower-fat vegetable oils with decreased serum HDL and LDL sizes might be due to a higher intake of SFA and a lower intake of PUFA. This is because sugary products are often sources of SFA, and lower-fat vegetable-oil-based fats are lower in PUFA content^(26)^. Considering the previously observed associations of lipoprotein particle sizes with cardiometabolic health^(48–53)^, our present results underline the importance of good diet quality in improving lipoprotein metabolism and preventing cardiometabolic diseases since childhood.

We also observed a direct association of vegetable, fruit and berry consumption with the concentration of small LDL particles. It is possible that fructose intake from this food group affects serum lipoprotein profile as fructose intake has been observed to influence the concentration of small LDL particles in children^(57,58)^ However, due to the complexity of human metabolism, other possible factors could explain the observed findings.

### Diet quality and serum apolipoproteins

We did not find any associations of diet quality with serum apolipoproteins from childhood to adolescence. We previously observed a cross-sectional association between a higher consumption of vegetables, fruit and berries with higher serum apoB/apoaA1^(19)^, but no such association was found in the present longitudinal study.

### Study strengths and limitations

We examined the longitudinal associations of diet quality with serum metabolites in a relatively large population sample of children followed for 8 years until adolescence using data on all these variables from three-time points. Another strength of our study is that most participants of this population-based sample of children followed up until adolescence have not been exposed to possible confounding factors, such as alcohol consumption, smoking, chronic diseases and medications. Additionally, we were able to control for sex, age, body fat percentage, pubertal status and physical activity, which were assessed at all three-time points. Food consumption was assessed comprehensively by 4-day dietary records reviewed by clinical nutritionists, thus improving the accuracy and reliability of the dietary data from the records. Overall diet quality was assessed using FCHEI, which has been validated in Finnish children^(28)^. To assess serum biomarkers, we used high-throughput NMR spectroscopy analysis which is a robust technique with multiple advantages in metabolomic research^(59)^. We also acknowledge the limitations of this study. First, the assessment of diet is always prone to misreporting^(60)^. Diet was reported by the parents or caregivers of the children at the baseline and 2-year examinations but by the adolescents themselves at the 8-year examinations, which might result in differences in reporting. For example, adolescents’ perceptions of their body image can lead to the misreporting of their diet^(61)^. Additionally, FCHEI has been validated in Finnish children but not adolescents. Due to the complexity of eating behaviour and human metabolism, it was not possible to consider all possible confounding factors in the statistical analyses. Another limitation of this study is the loss of 50 % of the original sample over 8 years. While those who participated in the 8-year examinations did not differ from non-participants in terms of baseline age, BMI-SDS, sex distribution or study group allocation, this level of attrition still reduces statistical power and may limit the overall generalisability of the findings. However, linear mixed-effects models used in our study for the analyses are especially suitable for analysing longitudinal datasets containing unbalanced data as the mixed-effect models assume that the data are missing at random. Finally, we studied Finnish children and adolescents, and the results are therefore not generalisable to all population groups. Thus, the interpretation of the present results should be done carefully.

### Conclusions

We observed longitudinal associations of overall diet quality and single indicators of diet quality with serum metabolites in a general population of children followed up until adolescence. Better diet quality was associated with a serum metabolite profile characterised by higher PUFA, lower SFA, BCAA and AAA as well as a smaller VLDL particle size, independent of body fat percentage, pubertal status and physical activity. The findings of this study are particularly relevant given the existing dietary challenges within Finnish children and adolescents^(20–22)^, among whom a low consumption of vegetables, fruit and berries and a higher consumption of dietary SFA and sugary products than recommended remains a concern. Thus, addressing these issues is fundamental for improving metabolic health in children and adolescents and building a base for precision nutrition^(62)^. These results suggest that healthy dietary choices since childhood modify the serum metabolite profile towards a more favourable direction for cardiometabolic health moving on to adolescence.
